# Screening of pesticide residues in soil and water samples from agricultural settings

**DOI:** 10.1186/1475-2875-5-22

**Published:** 2006-03-24

**Authors:** Martin C Akogbéto, Rousseau F Djouaka, Dorothée A Kindé-Gazard

**Affiliations:** 1 Centre de Recherche Entomologique de Cotonou, 06 BP 2604, Bénin; 2 Faculté des Sciences de la Santé, Université d'Abomey-Calavi, Bénin

## Abstract

**Background:**

The role of agricultural practices in the selection of insecticide resistance in malaria vectors has so far been hypothesized without clear evidence. Many mosquito species, *Anopheles gambiae *in particular, lay their eggs in breeding sites located around agricultural settings. There is a probability that, as a result of farming activities, insecticide residues may be found in soil and water, where they exercise a selection pressure on the larval stage of various populations of mosquitoes. To confirm this hypothesis, a study was conducted in the Republic of Benin to assess the environmental hazards which can be generated from massive use of pesticides in agricultural settings.

**Methods:**

Lacking an HPLC machine for direct quantification of insecticide residues in samples, this investigation was performed using indirect bioassays focussed on the study of factors inhibiting the normal growth of mosquito larvae in breeding sites. The speed of development was monitored as well as the yield of rearing *An. gambiae *larvae in breeding sites reconstituted with water and soil samples collected in agricultural areas known to be under pesticide pressure. Two strains of *An. gambiae *were used in this indirect bioassay: the pyrethroid-susceptible Kisumu strain and the resistant Ladji strain. The key approach in this methodology is based on comparison of the growth of larvae in test and in control breeding sites, the test samples having been collected from two vegetable farms.

**Results:**

Results obtained clearly show the presence of inhibiting factors on test samples. A normal growth of larvae was observed in control samples. In breeding sites simulated by using a few grams of soil samples from the two vegetable farms under constant insecticide treatments (test samples), a poor hatching rate of *Anopheles *eggs coupled with a retarded growth of larvae and a low yield of adult mosquitoes from hatched eggs, was noticed.

**Conclusion:**

Toxic factors inhibiting the hatching of anopheles eggs and the growth of larvae are probably pesticide residues from agricultural practices. Samples used during this indirect assay have been stored in the laboratory and will be analysed with HPLC techniques to confirm hypothesis of this study and to identify the various end products found in soil and water samples from agricultural settings under pesticide pressure.

## Background

The resistance of vectors to insecticides is a real handicap to the use of insecticide-treated materials. The recurrent presence on the agenda of most entomological research in Africa of vector resistance, more specifically of *Anopheles gambiae *resistance to insecticides, is due to the fact that insecticide-treated materials remain the principal tool of National Programmes of Malaria Control (NPMC) in the fight against vectors. The first cases of resistance were mentioned in the 1950s and 1960s with *An. gambiae *to organochlorine. Then, the phenomenon was limited to dieldrin and hexachlorocyclohexane (HCH) [1]. In Africa, the first cases of dieldrin resistance in *An. gambiae *were recorded in Burkina Faso in 1960 [2]. Ten years later, the identification of dieldrin resistance and cases of DDT resistance were reported in Togo, Senegal, Nigeria, Cameroon and Guinea [3]. There is no relation between DDT resistance and dieldrin resistance.

In the case of pyrethroids, resistance is mentioned relatively late, in the 1990s. The first cases of pyrethroid resistance were recorded in Côte d'Ivoire [4] and many other cases have been described in Kenya [5], Benin [6,7], Burkina Faso [7,8], Côte d'Ivoire [7] and Mali [9]. In central Africa, cases of pyrethroid resistance have been described in Cameroon and in the Central African Republic [10].

Despite the lack of concrete evidence, the use of insecticides in households and of pesticides in agricultural settings has been highlighted as a key factor contributing to the emergence of vector resistance. Some believe that resistance probably arose from the use of insecticide aerosols in households and some plants used for fumigation over a long period in rural and urban areas. In Benin Republic, Akogbeto and Yakoubou [6] suspect the emergence of DDT resistance, recorded in *An. gambiae *from meridian regions, to be related to two phenomena: (i) the massive use of DDT and dieldrin for house-spraying applications in southern villages from 1953 to 1960 during WHO programmes of malaria eradication [11] and (ii) the massive use of organochlorine in agricultural settings during the 1950s [3]. However, the absence of conclusive data regarding the implication of house-spraying in the selection of resistance seems to corroborate the observation of Mouchet [12], that no case of resistance has been recorded following the DDT house-spraying programmes performed over a period of 10 years in Madagascar, Thailand and South America.

Others believe that the emergence of resistance results from massive use of insecticides against pests in agricultural plantations. Recent studies conducted by Diabate *et al *[13] highlight the elevated levels of resistance genes, *kdr*, in *An. gambiae *collected in cotton-growing areas and constantly subjected to insecticide treatments, as compared to the low frequency of *kdr *recorded in rural areas where farmers are restricted to food crops for local consumption with no pesticides. In Côte d'Ivoire, the kdr mutation identified in resistant strains of *An. gambiae *was probably selected as a result of the massive use of DDT and pyrethroids against pests in cotton fields [7,8]. The hypothesis of a relation between some agricultural practices and the emergence of resistance should not be neglected. In Benin, insecticide treatments against pests in cotton plantations are done twice each month, for an average of three months (between July and October) each year. These treatment periods coincide with the rainy seasons and correspond to the period of high mosquito densities and increased development of *Anopheles *larvae. In vegetable farms, treatments are more regular and are done throughout the year. Pesticide treatments release active components into the environment of which some get directly into the breeding sites of mosquitoes. There is a high probability that insecticide residues can be found on soil in agricultural areas and could exercise a selection pressure on the larval stage on some populations of mosquitoes. To confirm this hypothesis, a study was carried out to assess environmental hazards related to the use of pesticides in agricultural settings. The study is a biological evaluation to screen residual insecticides on soil and water samples from vegetable farms subjected to pesticide treatments.

## Materials and methods

### Study area

This study was conducted in Benin Republic, West Africa. Two "test sites" and a "control site" were investigated. The two test sites were vegetable farms (Houeyiho and Parakou). The vegetable area of Houeyiho is a big farm of 14 hectares in the town of Cotonou. In Houeyiho, more than 300 farmers are involved in the cultivation of a large variety of vegetables: cabbages, carrots, lettuces, amaranth, cucumber etc. Farming at Houeyiho is associated with the use of insecticides to fight pests. The Parakou vegetable farm is located in the town of Parakou. This farm has similar characteristics to that in Houeyiho, but it is smaller in size and is less well-maintained. The control site selected for this study was the backyard of the Centre for Research in Entomology, Cotonou (CREC) located at Akpakpa, a peripheral locality of Cotonou. This site has a watering pool and its soil texture resembles that of the test sites. The main difference between the control site and the two test sites is the absence of insecticide pressure at the CREC premises. A recent study conducted in the test sites (Akogbéto *et al*, in press) confirmed the reality of the use of insecticides in these agricultural settings. Pyrethroids, and more specifically deltamethrin and cyfluthrin, are frequently used in the vegetable farms of Parakou and Houeyiho.

### Determination of levels of resistance of *Anopheles *populations from the 2 study sites

Prior to the evaluation of insecticide residues, the susceptibility to insecticides of *An. gambiae *samples from the two test sites was determined. The test was based on WHO standard protocols with two pyrethroids (permethrin 0, 75% and deltamethrin 0, 05%) and one organochlorine (DDT 4%). The use of pyrethroids and organochlorine in this test aimed to verify the presence of cross-resistance between the two families of insecticides. Mosquitoes exposed to insecticide papers in this test were three- to five-day old females of *Anopheles*, having emerged from larvae collected on the premises and in water spots found in the vegetable plantations of Houeyiho and Parakou. After exposure, dead and live *Anopheles *were separately kept on silica gel for further analysis of molecular forms and the identification of resistance genes (*kdr*). Total DNA extraction was conducted using modified techniques of Collins *et al *[14]. Amplification of DNA fragments were based on PCR techniques using appropriate primers, as described by Scott *et al *[15], for the identification of species of the *An. gambiae *complex, that of Favia *et al *[16] for molecular M/S forms, and that of Martinez Torres *et al *[17] for *kdr *mutations. Amplified fragments were migrated in electrophoresis tanks and bands were visualized under UV lights

### Protocol for biological evaluation of the presence of insecticide residues in soil and water samples from selected sites

Not having an HPLC machine for direct quantification of insecticide residues in collected samples, the protocol presented does not enable an identification and a quantification of insecticide residues in analysed samples. An indirect bioassay focussed on the study of factors capable of inhibiting the normal growth of mosquito larvae in breeding sites. Developmental speed and the yield of rearing *An. gambiae *larvae in breeding sites reconstituted with water and soil samples collected in agricultural areas under pesticide pressure, were monitored. Two strains of *An. gambiae *were used in this indirect bioassay: the pyrethroid-susceptible Kisumu strain and the resistant Ladji strain. *An. gambiae *Ladji was selected from the locality of Ladji at 5 km from Cotonou. The *Anopheles *population was purely "M" form, the level of susceptibility to permethrin and DDT was respectively 65% and 50%. It was in homozygous form, with an allelic frequency for the *kdr *mutations of one [6,7].

The protocol used in this evaluation is mainly based on comparison of the growth of larvae in test breeding sites (water and soil samples from agricultural settings under pesticide pressure) and in control breeding sites (water and soil samples from similar areas but not under pesticide pressure). Soil and water samples collected in the field were taken to the insectary where they were used for various simulations. Samples from Houyeiho and Parakou underwent series of simulations. The first set of breeding sites were reconstituted with top soil from vegetable farms mixed with water from control sites or mixed with watering water collected in the farm. The second set of breeding sites were made with soil collected in watering pools found in both farms (Houeyiho and Parakou) and used for the irrigation of vegetables. The control site selected for this study is the backyard of the Centre for Research in Entomology, Cotonou (CREC). This site has a watering pool and soil and water samples collected from CREC were closely similar to collections from study sites, the only difference being the absence of insecticide pressure at the CREC. Water from CREC was designated as CREC-water, whereas soil samples were designated as CREC-soil. The volume of water used in the reconstitution of breeding sites was 1,000 ml. For breeding sites made with a mixture of soil and water samples, 100 g of soil is well mixed in 1,000 ml of water.

An average of 200 eggs of the susceptible Kisumu strain was inoculated in each artificial breeding site. A similar inoculation was repeated with the resistant Ladji strain and for the different types of artificial breeding sites simulated. More than 4,000 eggs were inoculated and monitored during this biological evaluation. Monitoring of the growth of inoculated eggs led to assessing variations in hatching rates of eggs, speeds of development of larvae and yields of rearing larvae to adult mosquitoes with each strain of mosquito and each type of artificial breeding site. Data from breeding sites suspected to be contaminated with insecticide residues were compared with those from control sites. This comparison led to the confirmation of the potential presence of factors inhibiting the development of larvae. During this follow-up experiment, larvae in all artificial breeding sites were fed with similar quantity and type of food (well-ground cat biscuits mixed with yeast powder).

## Results

### Level of susceptibility of *Anopheles *populations from the three selected sites

A total of 775 females of *An. gambiae *were exposed to various types of impregnated papers for their levels of susceptibility prior to biological evaluations of pesticides. Results obtained were in line with past data published by Akogbeto and Yakoubou [6] and Akogbeto *et al *[18] in the same localities. Data recorded highlight elevated resistance to permethrin at 0.75% and DDT at 4% in the locality of Houéyiho (Table 1). In the vegetable farm of Parakou, *Anopheles *species collected were susceptible to pyrethroids despite the use of insecticides in the locality against pests. These results could be explained by abundance of populations of *An. arabiensis *(87.5%) in the area. However, a reduced susceptibility to DDT was recorded at 4% (mortality rate = 90%) with *An. gambiae *from Parakou. In general, susceptibility tests conducted in this study were confirmed with PCR analysis performed in the two sites. High frequencies of *kdr *mutations were recorded at Houéyiho (90%) and low frequencies at Parakou 10%)(Table 1).

**Table 1:** Susceptibility tests and PCR analysis on *Anopheles *populations from vegetable areas of Houeyiho and Parakou

Laboratory processing	Houeyiho			Parakou		
	Perm.0.75%	Delta 0.05	DDT 4%	Perm.0.75%	Delta 0.05	DDT 4%
**Mosquitoes exposed to insecticides**	120	100	130	80	80	80
% Mortality	70	94	70	96	100	90
**Mosquitoes tested for PCR**	30	-	-	80	-	-
*An. gambiae s.s.*	30	-	-	10	-	-
*An. arabiensis*	0	-	-	70	-	-
Freq. M	100	-	-	40	-	-
Freq. S	0	-	-	60	-	-
Freq. Kdr	90	-	-	10	-	-

### Impact of agricultural treatments with insecticides on hatching rates of *An. gambiae *eggs

**Figure 1:**
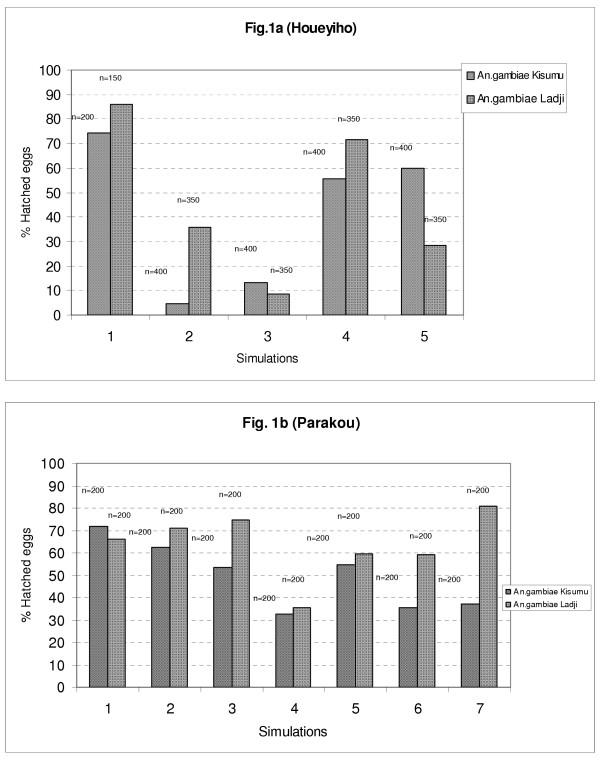
**Hatching rates of eggs of 2 strains of *An. gambiae *in simulated breeding sites using samples of soil substrates and water from vegetable areas of Houeyiho and Parakou. **1. Control: CREC-water. 2. Superficial soil from ridges in vegetable areas + CREC- water. 3. Superficial soil in between ridges in vegetable areas + CREC- water. 4. Soil from watering pools + Water from watering pools. 5. Soil from watering pools + CREC-water. 6. Water from watering pools alone. 7. Big water pool found exclusively at Parakou farm. n : number of eggs inoculated in each simultation.

The CREC-water taken alone offers favourable conditions for hatching of *An. gambiae *eggs. When few grams of soil samples from agricultural areas under insecticide treatments are added into it, a decrease in hatching rates is observed. The mean hatching rate recorded in control breeding sites is 74.5% (n = 200) for the susceptible Kisumu strain and 86% (n = 150) for the resistant Ladji strain. With breeding sites reconstituted with soil and water samples from the vegetable farm of Houeyiho, a significant drop in hatching rates was recorded with both strains: 33.4% (n = 400) and 36.7% (n = 350), for Kisumu and Ladji respectively (*P*-Kisumu = 0.00, X^2 ^= 89.9, df = 1: *P*-Ladji = 0.00, X^2 ^= 102.69, df = 1). A similar observation was made with breeding sites from the Parakou vegetable farm. Here, the hatching rate of the Kisumu strain dropped from 68.7% (n = 200) in the corresponding control sample to 45.9% (n = 200) in the test sample (*P *= 0.00005, X^2 ^= 20.6, df = 1). However, with the resistant Ladji strain, similar hatching rates were recorded with control samples (62.2%: n = 200) and samples suspected to contain insecticide residues (63.4%: n = 200), (*P *= 0.75, X^2 ^= 0.1, df = 1) (Figure 1).

In other simulations, the decrease in hatching rate was spectacular. In Houeyiho, for example, artificial breeding sites formulated with top soil and water from watering pools gave hatching rates as low as 13.2% with the Kisumu strain and 8.5% with the Ladji strain compared to 74.5% and 86% in control breeding sites, with both strains respectively (Figure 1a).

### Impact of agricultural treatments with insecticides on development of *An. gambiae *larvae

**Figure 2:**
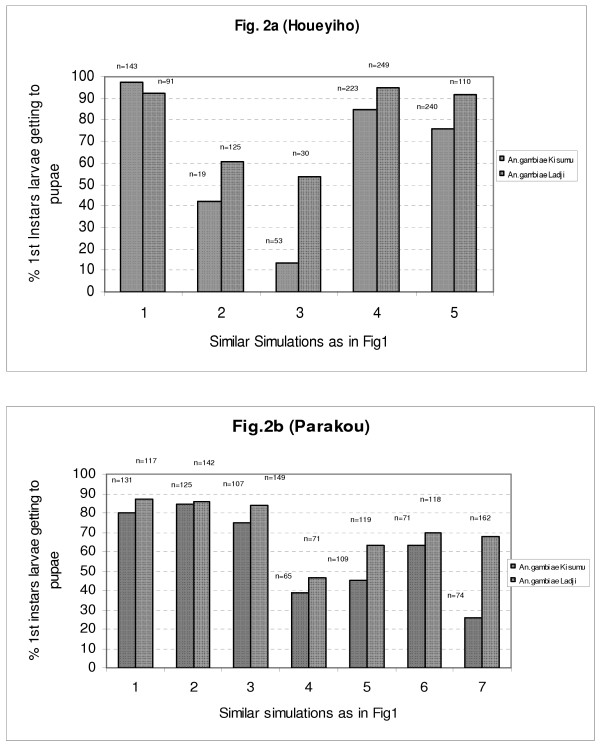
Rate of 1st instars larvae getting to pupae stages in simulated breeding sites using samples of soil substrates and water from vegetable areas of Houeyiho and Parakou.

The development of larvae was estimated by quantifying the proportion of first instars larvae reaching the pupae stage. This is the best indicator to monitor when evaluating factors inhibiting larval growth. Contrary to the first indicator, which is based on fast hatching process of eggs, this second indicator takes into consideration the developmental cycle of larvae from first instars to pupae. This cycle is relatively long and involves many interactions between larvae and constituents of breeding sites which allows, therefore, a good follow-up of expressions of inhibiting factors in simulated breeding sites. In control breeding sites, 84.7 to 99.3% of first instars larvae of the Kisumu strain were able to reach pupae stage, giving a mean of 97.2%. Similar figures were also recorded with the resistant Ladji strain in controls (mean of 92.3%)(Figure 2). In breeding sites constituted of samples from agricultural settings under insecticide pressure, breeding of larvae gave very low rates of first instars larvae reaching the pupal stage: 13.2% (n = 53) was recorded with Kisumu strains in breeding sites made from combinations of top soil and water from watering pools of the Houeyiho vegetable farm. With the same strain, 42.1% of larvae reaching the pupal stage were recorded in breeding sites from mixtures of top soil from Houeyiho and CREC-water. With the Ladji strain, although the results obtained in test samples were low compared to controls (92.3%), a higher rate of Ladji larvae reaching pupal stage was recorded with the two simulations: 53.3% (n = 30) and 60.8% (n = 125), respectively (Figure 2a). Cumulative analysis of results recorded with the development of both strains of *An. gambiae *on artificial breeding sites from agricultural settings showed that Ladji larvae were less susceptible to presumed toxic breeding sites (75% reaching the pupae stage) compared to 53.9% of Kisumu larvae (*P *= 0.0064 X^2 ^= 7.41, df = 1). Similar observations were made with samples from vegetable areas of Parakou. Simulations from Parakou produced a relatively low development of larvae in presumed toxic breeding sites (55.3% for Kisumu strain: n = 92 and 87.2% for Ladji strain: n = 117) compared to controls (75.5%: n = 138 for Kisumu and 83% n = 125 for Ladji) (*P*-kisumu = 0.0015, X^2 ^= 9.9, df = 1: *P*-ladji = 0.38, X^2 ^= 0.76, df = 1)(Figure 2).

### Impact of agricultural treatments with insecticide on the yield of rearing *An. gambiae*

**Figure 3:**
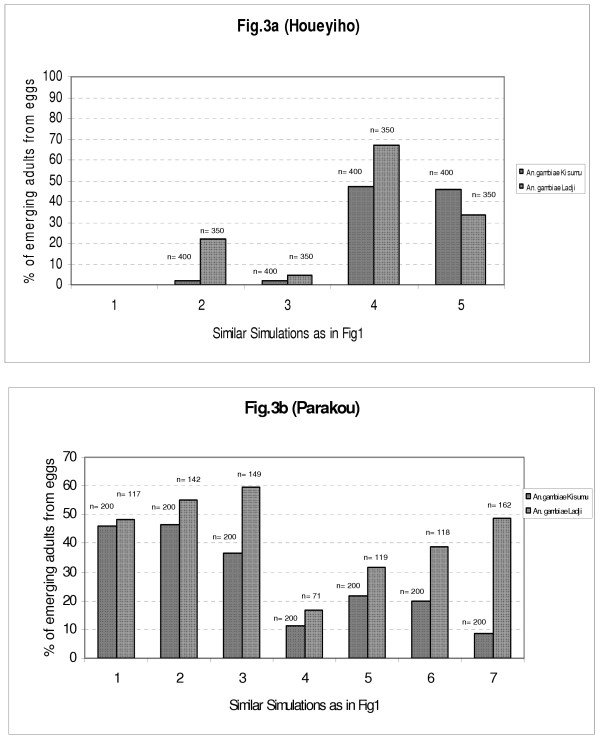
Yield of rearing to adults of *An. gambiae *eggs in simulated breeding sites using samples of soil substrates and water from vegetable areas of Houeyiho and Parakou.

This yield expresses proportions of *An. gambiae *eggs reaching adult stage. This is also a good indicator to assess toxicity of breeding sites in which eggs were inoculated. Results globally obtained with this indicator were not spectacular, with low figures obtained even with controls: 45% with the Kisumu strain and 48.5% with the Ladji strain. When eggs were inoculated into artificial breeding sites from agricultural areas, the yield of adult mosquitoes was reduced by half. Comparisons made with both strains throughout the experiment showed that the Kisumu strain faced more difficulties in simulated breeding sites than the Ladji strain. The growth of the Ladji strain was less affected by simulations than the Kisumu strain (Figure 3).

When eggs of both strains were grown in artificial breeding sites reconstituted with soil and water samples from the two agricultural areas selected in this study, the yield of resistant strains was always high compared to susceptible strains: 31.8% (n = 350) versus 24% (n = 400) in Houeyiho (*P *= 0.01, X^2 ^= 5.56, df = 1)(Figure 3a) and 62.6% (n = 200) versus 24.1% (n = 200) in Parakou (*P *= 0.00000, X^2 ^= 69.39, df = 1)(Figure 3b).

## Discussion and conclusion

The results of this study suggest that samples from agricultural settings under pesticide pressure contain inhibitory factors responsible for the reduced growth rate in larvae of *Anopheles *Kisumu, with a lesser inhibitory effect on the development of the resistant Ladji strain.

Parameters targeted during this study have been analysed and interpreted separately according to their respective values. Because of the short duration of hatching process with *Anopheles *eggs, the hatching rate should be considered as a simple signal of toxicity of samples from agricultural settings. The rapid hatching of eggs when introduced in simulated breeding sites does not give room for expression of inhibitory factors on embryos. This hypothesis probably explains similarities in data recorded in Parakou, where hatching rates of the resistant Ladji strain did not appear to be influenced by artificial breeding sites generated with water from watering pools of Parakou and those from top soil mixed with CREC-water (63.4% and 62.2% respectively for the two sets of artificial sites).

### Anopheles

Ladji is a strain selected from a relatively polluted locality, the locality of Ladji in peripheral region of Cotonou. This strain had probably developed over time some capacity to withstand low levels of toxicity. This assumption could explain the low inhibitory impact of breeding sites on hatching of *Anopheles *Ladji eggs recorded in this study.

As observed with hatching rates, larval development also varies with respect to strains of *Anopheles *inoculated and types of artificial breeding sites simulated. In the vegetable farm of Houeyiho, breeding sites simulated with top soil collected around vegetables seemed to inhibit larval growth more than simulations with watering water and soil from watering pools. In these two simulations, inhibitory effects are less spectacular. In breeding sites generated with water mixed with soil from watering pools, 84.7% of *Anopheles *Kisumu eggs were able to reach the pupal stage, whereas, in simulations with water mixed with soil collected around vegetables, only 13.2% of larvae were able to reach the pupal stage. This consistent difference in results suggests an unequal distribution of insecticide residues after treatment in vegetable farms of Houeyiho. A similar trend was recorded with data from vegetable areas of Parakou. However, in Parakou, simulations using irrigation water collected from cultivated areas seem more toxic compared to other simulations. A recent study conducted by Akogbeto *et al *[18] reveals that several chemicals are used in the vegetable farms of Houeyiho and Parakou against field pests. These chemicals are mainly pyrethroids, organophosphates and carbamates. These compounds are used as single formulations or as combinations of two or three insecticides of different families, the final aim being to generate a synergy of insecticides for a better pest management. After pesticide treatments in agricultural settings, residues of insecticides get into mosquito breeding sites. These residues have lethal effects on larvae of some populations of mosquito whereas they exert a selective pressure on other populations, leading to a gradual tolerance of insecticide concentrations and to the emergence of resistant populations.

Insecticides used in public health against disease vectors are similar to those used for years in agriculture. In Benin Republic, pyrethroids were introduced in agriculture in the 1970s and, after 30 years of continuous use, cases of resistance may be found in some populations of insects. With regard to the origins of vector resistance identified in rural and urban areas, various diverging hypothesis are put forward. Some authors incriminate pesticides used in cotton farms and rice fields as the main source of selection of resistance in several species of mosquito in rural environments (Georghiou [19] in Central America, Asia and Africa; Chandre *et al. *[7] and N'guessan *et al*. [20] in Côte d'Ivoire; Diabate *et al*. [15] in Burkina Faso). The important movement of young people from villages to towns, as a result of unemployement, has led to the development of agricultural spaces within urban areas, where vegetables are intensely cultivated. These vegetable farms found in urban settings are active throughout the year because of constant and high demands of urban populations. To keep their productivity high and avoid shortages of vegetables in urban areas, farmers are forced to treat their farms at relatively high frequencies. This condition explains the increased use of insecticides in urban centres as opposed rural areas. Pyrethroids in contact with water undergo a rapid colloidal transformation, followed by sedimentation. After pesticide treatment, insecticide residues are washed by rainfall and sediment in watering pools located below cultivated areas. This is probably what happens in Parakou, in view of the sloping terrain where the vegetable farm is located. The terrain allows a fast sweeping of pesticide residues down the slope and could explain the high levels of toxicities recorded in watering pools located below cultivated sections compared to top soil collected on cultivated sections. In addition to this washing process, insecticide residues generated by agricultural practices are also degraded by strong sunrays recorded in this northern part of Benin Republic. At Houeyiho, the washing phenomenon also exists but is relatively low compared to the frequent and massive use of insecticides. This insecticide pressure keeps the level of insecticide residues in the soil high and may explain the elevated toxicity observed with top soil. Data from this study indicate that factors inhibiting the hatching of *An. gambiae *eggs and the development of their larvae are insecticide residues from agricultural practices. Some samples of soil and water from the three study sites were stored in the laboratory and will be analysed by HPLC to verify the hypothesis and to identify the chemical compounds present in water and soil samples.

## Authors' contributions

MCA conceived the study and participated in data interpretation and manuscript preparation. RFD carried out the study design, sample processing and data analysis. DAKG participated in the design of the study and substantially helped draft the manuscript.
